# Marginal zone lymphomas in HIV patients

**DOI:** 10.1186/1742-4690-9-S1-P135

**Published:** 2012-05-25

**Authors:** Philippe Genet, Christine Fourcade, Virginie Masse, Bouchra Wifaq, Laurent Sutton, Dris Chaoui, Ahmad Al Jijakli, Nina Arakelian

**Affiliations:** 1 Hématologie at Centre Hospitalier Victor Dupouy, Argenteuil, France

## 

Incidence of Non-Hodgkin’s Lymphoma (NHL) remains high in the context of HIV infection. High-grade NHL are the sub-types commonly seen. Indolent lymphomas have been rarely described although several cases of follicular or marginal zone lymphoma (MZL) have been published. Among MZL, MALT lymphomas are predominant. We describe 3 cases of MZL with leukemic presentation.

In our on-going cohort of 580 HIV-patients, 3 cases of MZL were identified. The following characteristic were recorded: clinical exam, thoracoabdominal CT-scan, cytological aspect of the peripheral blood smear, immunologic and cytogenetic analysis.

There were 2 male and 1 female. Duration of HIV infection was 8, 13 and 18 when MZL was diagnosed. HCV serology was positive in 2 cases with pcr negativity in one case. In all cases, MZL was diagnosed during the occurrence of a mild hyperlymphocytosis: 5000, 6000 and 9000/mm3 respectively while hemoglobin and platelets remained normal. All patients were received HAART for more than 8 years and all have CD4 above 500/mm3 and viral load under 50 copies/ml. All patients were asymptomatic without peripheral adenopathy neither hepatosplenomegaly. On thoracoabdominal CT scan, only infracentrimetric adenopathy were detected. On peripheral smears, several lymphocytes with a villous aspect were detected for each patient. In all cases, immunologic phenotype by FACS was consistent with the diagnosis of MZL. Karyotype of the peripheral lymphocytes was normal in one case, revealed an isochromosome 3 in the second case and showed 46, XX, der(1)t(1;?), t(8;14;18)(q24;q32;q21) in the last case. After a follow-up of 2, 2 and 5 years, all patients are alive with a stable disease without chemotherapy.

These data suggest that, as for non-HIV infected patients, MZL have an indolent course in HIV patients.

